# MTL_TX: A Multi-Task Transformer Model for Improved Radiation Time-Series Estimation

**DOI:** 10.3390/s26051439

**Published:** 2026-02-25

**Authors:** Hongfang Zhang, Adam Stavola, Hal Ferguson, Bence Budavari, Hongyi Wu, Chiman Kwan, Jiang Li

**Affiliations:** 1 Department of Electrical & Computer Engineering, Old Dominion University, Norfolk, VA 23529, USA; hzhan005@odu.edu (H.Z.); astav002@odu.edu (A.S.); hferg003@odu.edu (H.F.); 2 Thomas Jefferson National Accelerator Facility, Newport News, VA 23606, USA; 3 Applied Research LLC, Rockville, MD 20850, USA; bencebudavari@gmail.com; 4 Department of Electrical & Computer Engineering, University of Arizona, Tucson, AZ 85721, USA; mhwu@arizona.edu; 5 Johns Hopkins University Applied Physics Laboratory, Laurel, MD 20723, USA; chiman.kwan@arllc.net

**Keywords:** radiation dose estimation, multi-task learning, Transformer

## Abstract

Controlling radiation doses at potential radioactive facilities is critical to ensuring the safety of both personnel and the public. At the Thomas Jefferson National Accelerator Facility (JLab), multiple sensors are deployed around the three experimental halls to monitor key parameters, including single-beam current, energy levels, current leakage, and radiation values during accelerator operations. In this study, we developed a Multi-task Transformer model, MTL_TX, to accurately estimate radiation doses at sensor locations based on historical data, with the aim of enhancing safety in accelerator facilities and surrounding public areas. To improve estimation accuracy, we integrated two innovative components into the proposed model: hierarchical feature embedding (HFE) and multi-level decomposition attention (MDA). Additionally, the multi-task learning (MTL) framework effectively leverages correlations among multiple sensors, enabling individual estimations for each sensor. MTL_TX achieved outstanding results on data collected in 2018, with an MSE of 0.1464, an RMSE of 0.2353, and an $R^{2}$ score of 0.8584. Furthermore, when trained on 2018 data, MTL_TX exhibited excellent generalization capability to unseen datasets from 2016 to 2019, achieving an MSE of 0.1407, an RMSE of 0.2263, and an $R^{2}$ score of 0.8831. These results demonstrate a significant improvement over existing state-of-the-art models.

## 1. Introduction

Radiation estimation is a critical aspect of controlling radiation dispersion in accelerator facilities. The Department of Energy (DOE) mandates that radiation exposure in such facilities complies with legal standards to protect personnel, the public, and the environment [1]. At the Thomas Jefferson National Accelerator Facility (JLab), administrative control policies are implemented to maintain radiation exposure as low as reasonably achievable (ALARA), limiting annual doses for unmonitored personnel and the public to 10% of the federal limit (0.1 mSv or 10 mrem). Therefore, minimizing radiation exposure for personnel within the facility without compromising operational efficiency is essential. Traditional methods of controlling radiation exposure primarily focus on controlling radiation sources and improving protective measures between sources and personnel. To ensure compliance with radiation safety regulations, facilities typically rely on active and passive real-time monitoring using radiation sensors to assess both on-site and off-site radiation fields. However, this approach often requires significant resources for effective radiation exposure management, creating challenges for optimizing safety and operational efficiency.

Deep learning (DL) models have demonstrated superior performance in radiation analysis across various radiation sources [2,3,4,5]. In our previous work, we developed a multi-task learning (MTL) framework based on accelerator data characteristics, utilizing long short-term memory (LSTM) and convolutional neural network (CNN) architectures as backbones for effective radiation estimation [6]. Leveraging DL models for automated radiation dose estimation reduces operational costs and enables real-time monitoring, ensuring regulatory compliance for accelerator configurations. Transformers [7] have been extensively studied and have proven to outperform traditional neural networks in time-series analysis, offering enhanced capabilities for capturing both short-term and long-term dependencies. However, there are no existing Transformer-based models specifically designed for radiation estimation in high-energy accelerator environments, which leaves a gap in benchmark comparisons. This paper aims to develop an MTL deep learning model, named MTL_TX, which is based on the Transformer architecture to enhance radiation estimation.

MTL_TX is designed to capture correlations among historical readings across all sensors and provide synchronized, real-time radiation estimates across multiple sensor locations. It is important to clarify that the proposed framework is not intended to model the underlying radiation physics or radiation transport mechanisms of accelerator facilities. Instead, this work addresses the problem of multi-sensor radiation estimation, where reliable real-time estimation must be achieved under noisy, incomplete, and heterogeneous sensing conditions. Building on existing innovations in Transformer architectures, we introduce novel components tailored to the unique characteristics of the collected data, with further enhancement of radiation estimation performance. The proposed model is comprehensively compared with previously developed deep learning frameworks and several other competing methods. Experimental results demonstrate that MTL_TX achieves state-of-the-art performance. Our main contributions are summarized as follows:

- Radiation monitoring at high-energy accelerator facilities is formulated as a multi-sensor estimation problem, and a unified Transformer-based multi-task framework is constructed to jointly estimate radiation values at multiple sensor locations.
- Two novel components, hierarchical feature embedding (HFE) and multi-level decomposition attention (MDA), are specifically tailored for radiation estimation.
- Extensive experiments demonstrate the superior performance of the proposed model, particularly in estimating radiation doses on unseen datasets.

The HFE component integrates global variate embeddings and local patch embeddings into a hierarchical representation to capture inter-sensor dependencies, and the MDA component decomposes input sequences into trend and seasonal components to model multi-level temporal patterns. In summary, MTL_TX achieved $R^{2}$ = 0.8584 and RMSE = 0.2353 on unseen data from the same year, and an average $R^{2}$ = 0.8831 with RMSE = 0.2263 across different years.

The remainder of this paper is organized as follows: Section 2 reviews the related work. Section 3 describes the proposed methodology. Section 4 outlines the experimental setup. Section 5 analyzes the experimental results. Section 6 discusses implications and limitations. Finally, Section 7 concludes the study and outlines directions for future research.

## 2. Related Work

### 2.1. Radiation Monitoring at JLab

**Figure 1:**
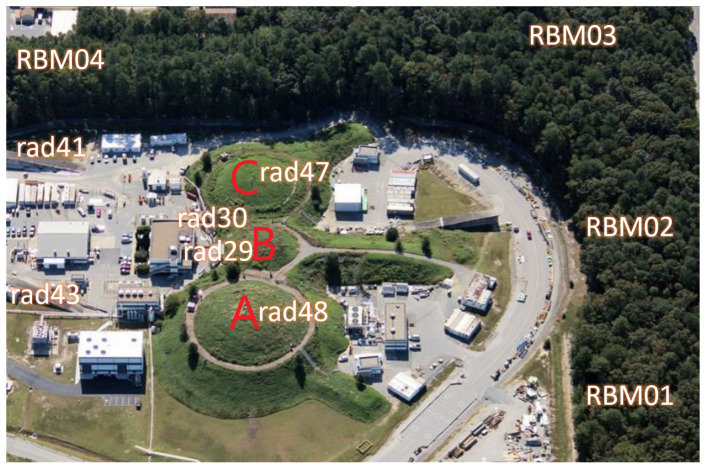
JLab campus with experimental halls “A”, “B”, “C” and radiation monitoring sensors. "rad##" and "RBM##" stand for on-site and boundary radiation sensors, respectively.

The main research facility at JLab is the continuous electron beam accelerator facility (CEBAF), which consists of a polarized electron source, an injector, and a pair of superconducting radiofrequency linear accelerators [8]. This facility is capable of accelerating electrons (negatively charged subatomic particles) to nearly the speed of light. The beam delivered to Halls “A”, “B“, and “C“ reaches approximately 11 GeV after five full passes [9]. The electron beam is delivered to four experimental halls, labeled “A”, “B“, and “C“, as shown in Figure 1. The facility is located in Newport News, Virginia. Each hall is equipped with specialized spectrometers to record the products of collisions between the electron beam or real photons and stationary targets; these interactions generate radiation exposure during operation.

Multiple radiation sensors are deployed around the experimental halls to monitor both accessible and non-accessible areas in real time. For instance, sensors labeled “rad##“ represent on-site monitors, while sensors labeled “RBM##“ indicate Radiation Boundary Monitors (RBMs), where “##“ denotes the specific location number. The boundary sensors play a crucial role in monitoring radiation exposure in public areas adjacent to the accelerator facility. Each on-site monitoring sensor is equipped with two channels: gamma and neutron, whose data are archived in the system as “_p1“ and “_p2“, respectively. This study focuses on analyzing the radiation doses in Hall “A”, utilizing data from nearby sensors “rad29“, “rad43“, and “rad48“. These sensors provide essential real-time radiation data, supporting both operational safety and regulatory compliance at JLab.

On-site gamma radiation sensors, referred to as Continuous Area Radiation Monitors (CARM), utilize commercially available probes (Canberra^®^/RemRad^®^ IP100 series ionization chambers) with a typical dose measurement range of 100 µSv/h to 1 Sv/h (0.1 mrem/h to 1 R/h). Neutron radiation is measured using BF3 probes inside Anderson–Braun-type moderators. The effective dose measured by these instruments quantifies the biological risk associated with specific radiation levels. Each radiation sensor independently measures neutron and gamma radiation, and the total radiation dose received by personnel on-site is calculated as the sum of these two components.

Radiation data are collected on a rolling basis with an interval typically not exceeding one minute. The instruments are equipped with internal settings to convert count rates into dose rates (e.g., µSv/h) and to store the collected dose rates in the JLab archival system. This system provides programmatic access to historical data spanning several years and facilitates temporal alignment of outputs from multiple sensors through data resampling. In this study, we focus on data archived from 2016 to 2019, with a resampled interval of one hour.

### 2.2. Transformer Framework

Transformer models have revolutionized deep learning by replacing traditional recurrent and convolutional operations with self-attention mechanisms [7]. This architectural shift enables efficient parallel computation and the capture of long-range dependencies, making Transformers highly effective in natural language processing, computer vision, and speech recognition. Recent advancements highlight their success across various domains, including BERT [10] and GPT-3 [11] in language modeling, Vision Transformer (ViT) [12] in image analysis, and Speech-Transformer [13] in audio processing. In time-series analysis, Transformers have gained attention for their ability to model complex temporal dependencies. Various studies have refined the Vanilla Transformer to address domain-specific challenges. Informer [14] addressed the inefficiencies of standard attention mechanisms by introducing sparse self-attention and a ProbSparse strategy, enabling long-sequence forecasting with reduced computational overhead. Autoformer [15] extended this approach by incorporating decomposition-based attention mechanisms, separating trend and seasonal components to improve interpretability and estimation accuracy. FEDformer [16] integrated frequency-domain modeling through Fourier and wavelet transforms, effectively capturing both local and global temporal patterns.

### 2.3. Time-Series Embedding

Recent advancements in embedding strategies have enhanced feature representation and inter-variable relationships in time-series models. PatchTST [17] segments each variable into non-overlapping patches, effectively capturing local temporal patterns. iTransformer [18] inverts the embedding dimension, mapping a single variable’s entire sequence into a high-dimensional feature space (Variate Tokens), allowing the attention mechanism to naturally model multivariate correlations. These studies improve the efficiency and scalability of Transformers in handling complex time-series data. Our previous work has preliminarily examined the impact of different embedding methods and attention mechanisms on JLab-specific tasks [19,20]. Transformers have also shown promise in specific applications such as anomaly detection [21], energy forecasting [22], and industrial IoT data analysis [23]. However, the application of Transformers to datasets in high-energy accelerator facilities remains unproven.

### 2.4. Multi-Task Learning

Multi-task learning (MTL) enhances machine learning models by leveraging shared representations across related tasks, improving generalization and reducing overfitting compared to single-task learning (STL) [24]. In time-series analysis tasks, MTL enables simultaneous learning from multiple correlated sequences. MTTrans [25] introduced a multi-task Transformer model with shared attention blocks and task-specific decoders to balance knowledge sharing and task differentiation. Crossformer [26] extended this approach with cross-dimension attention, capturing variable interdependencies while supporting multi-task predictions. Transformer models also perform well in cross-domain MTL by utilizing shared attention layers. Perceiver [27] leverages latent space representations to handle multi-modal and multi-task data, offering a flexible framework for diverse inputs. In healthcare, TransMTL [28] applies Transformers to jointly predict patient outcomes across multiple clinical tasks, demonstrating their ability to integrate task-specific nuances with shared temporal patterns.

## 3. Methodology

### 3.1. Data Pre-Processing and Selection

This study utilizes archived datasets collected at JLab from 2016 to 2019 and focuses on radiation sensors deployed near Hall A to address the challenge of real-time radiation estimation. Table 1 summarizes the radiation sensors deployed around Hall A, each monitoring distinct physical parameters: a sensor for injected current (labeled “IBC1H04CRCUR2”), a sensor for energy measurement (labeled “MMSHLAE”), a beam loss monitor (BLA, labeled “IBCBS05CLOSS”), and radiation sensors for monitoring gamma and neutrons (labeled “rad48”, “rad43”, and “rad29”). The data usage of each sensor in the model is also indicated in the table.

**Figure 2:**
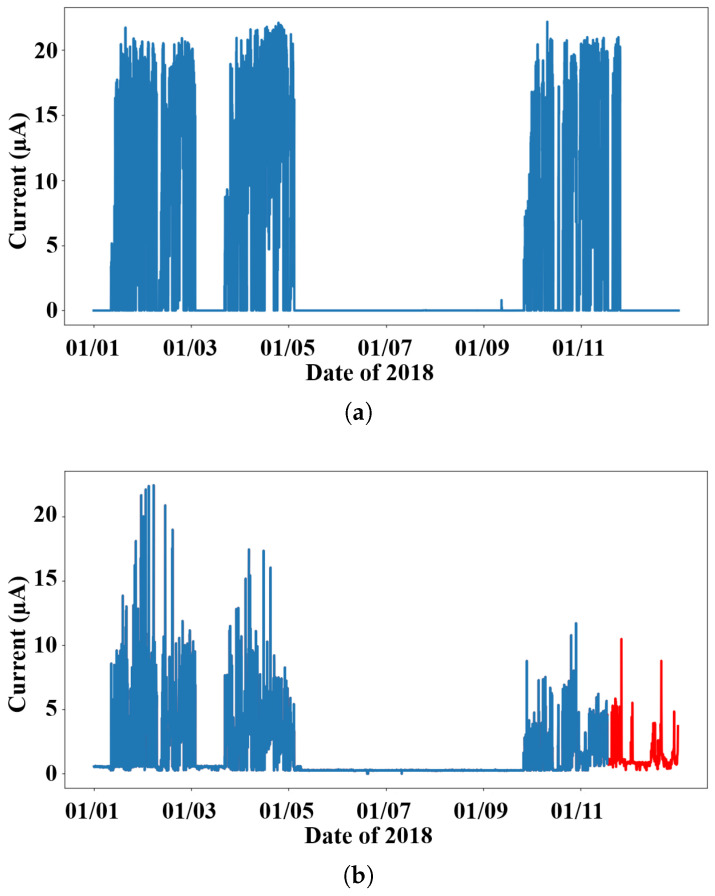
Hourly sample data near Hall “A” for the entire year of 2018. The red segment was deleted from modeling since the counterpart in the current series in (**a**) is missing. (**a**) Input current measured by the “IBC1H04CRCUR2” sensor. (**b**) Gamma radiation collected by the “rad48_p1” sensor.

Figure 2a presents the hourly injected beam current values for Hall A during the 2018 experimental period, while Figure 2b shows the gamma radiation recorded by the “rad48_p1” sensor above Hall A. The archived radiation data contains noise arising from missing beam-related measurements, sensor malfunctions, and operational transitions. To ensure physical consistency, radiation readings whose corresponding injected beam current is missing or unchanged were treated as invalid. Anomalous readings of the “rad48_p1” sensor at the end of 2018 have been removed due to their irrelevance to the corresponding beam current values, as highlighted in red in Figure 2b. Table 2 summarizes the distribution of data segments used in this study. Based on data continuity and noise conditions, where most remaining periods exhibit only near-zero fluctuations, segments were selected according to intervals containing sustained and physically meaningful radiation responses. The 2018 dataset was divided into three segments for model training and testing. The 2016, 2017, and 2019 datasets contained only one usable segment that satisfied these criteria due to higher noise levels and data discontinuities and were used to assess the model’s generalization capabilities.

**Figure 3:**
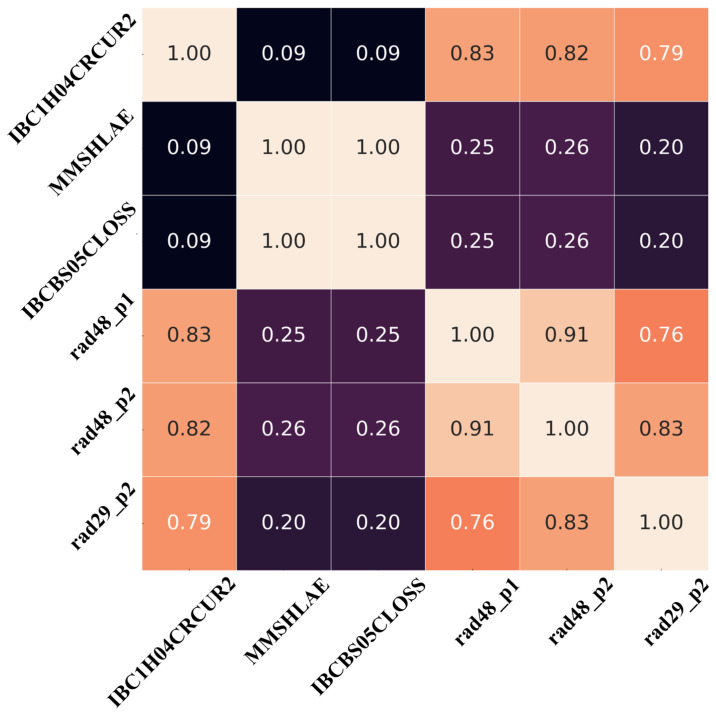
Correlation matrix among input current, energy, BLA and radiation values measured using different sensors.

The “rad43_p1” and “rad43_p2” sensors were excluded from this study due to excessive noise in their recorded data, while the “rad29_p1” sensor was excluded due to significant data gaps. Figure 3 illustrates the correlation matrix for sensor data recorded in Hall A during 2018, including injected single-beam current, energy, beam loss accounting (BLA), and radiation values. The matrix employs Pearson correlation coefficients [−1, 1] to quantify relationships between variable pairs: values near ±1 indicate strong correlations, while those close to 0 suggest weak or no linear dependency. The injected beam current shows a clear correlation with all radiation values, as it is the primary source of radiation. Although BLA exhibits a relatively low correlation with other sensor data, it measures beam current loss in the injector and provides an underlying physical link between current and energy. To estimate current radiation values at “rad48_p1”, “rad48_p2”, and “rad29_p2”, the model leverages historical and concurrent data from Hall A’s beam current, injected energy, BLA, and past radiation measurements.

**Figure 4:**
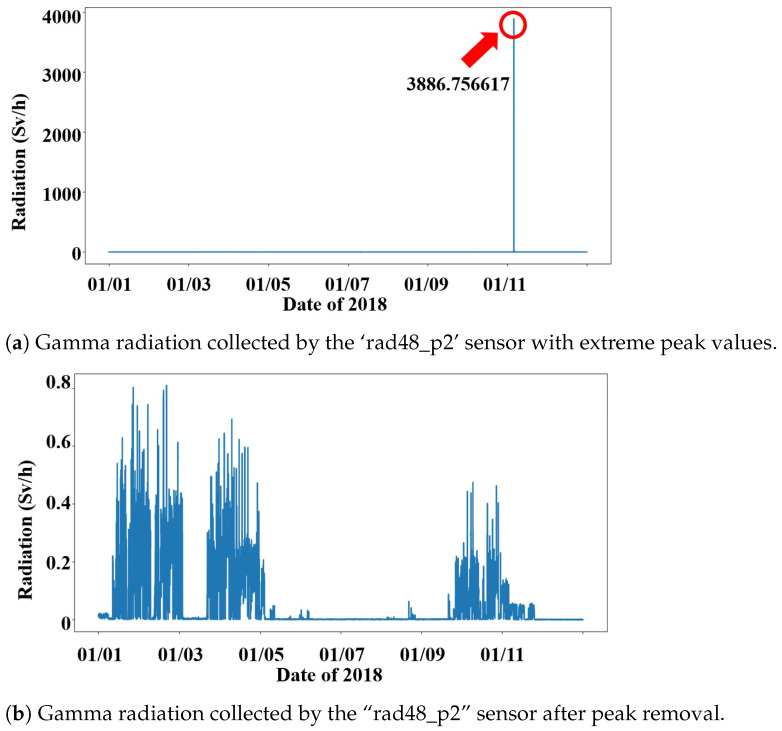
Hourly sample radiation data from the “rad48_p2” sensor near Hall “A” in 2018. (**a**) the original series containing extreme anomalous peaks, while (**b**) illustrates the radiation series after peak values were set to zero to preserve temporal continuity.

Radiation data collected in accelerator environments occasionally exhibits extreme spikes that exceed normal radiation levels by several orders of magnitude. As shown in Figure 4a, the “rad48_p2” sensor in 2018 contains multiple abnormal peaks, with the maximum reaching 3886.76 Sv/h. These values are physically implausible under normal beam operation and are not accompanied by corresponding changes in beam current or energy, indicating data logging errors. To mitigate their impact on model training while preserving temporal continuity, such peak values were not removed but set to zero. After this handling, the radiation series returns to a stable and physically reasonable range, as illustrated in Figure 4b. This strategy prevents extreme outliers from dominating the loss function and was applied consistently across all datasets.

### 3.2. Radiation Estimation

The proposed deep learning model takes historical sensor readings as input and outputs the estimated radiation values for the sensors at the current time step. Let $C={\left\{c_{t}\right\}}_{t=1}^{T}$, $E={\left\{e_{t}\right\}}_{t=1}^{T}$, and $B={\left\{b_{t}\right\}}_{t=1}^{T}$ represent the time series of injected current, energy, and beam loss accounting (BLA) recorded at JLab, respectively, where each sequence has a length of *T*. Similarly, let $R^{j}={\left\{r_{t}^{j}\right\}}_{t=1}^{T}$ denote the radiation time series recorded by sensors “rad48_p1”, “rad48_p2”, and “rad29_p2”, where *j* indexes the specific sensor and each sequence also has a length of *T*. The model estimates the radiation values aat the current time step, *t*, $r_{t}^{j}$, by leveraging the previous *m* time steps of data from *C*, *E*, *B*, and $R^{j}$. This task can be formulated using either single-task learning (STL) or multi-task learning (MTL) frameworks.

Before constructing supervised learning samples for radiation estimation, the raw sensor time series are processed through a deterministic preprocessing pipeline. This pipeline enforces physical consistency, removes spurious extreme values, and extracts contiguous beam-on segments suitable for model training and evaluation. The overall preprocessing procedure is summarized in Algorithm 1.
**Algorithm 1** Data preprocessing pipeline for radiation time-series modeling.1.**Input:** Raw multivariate time series ${\left\{C\left(t\right),E\left(t\right),B\left(t\right),R^{j}\left(t\right)\right\}}_{t=1}^{T}$, and signal-specific peak thresholds $\alpha =\{\alpha_{X}\}$.2.**Output:** Cleaned contiguous time series Γ for STL and MTL model training and evaluation.3.**Missing-data handling:** Construct a validity mask$$M\left(t\right)=1_{C(t)>0}\cdot 1_{E(t)>0}\cdot 1_{B(t)>0}.$$All time indices with M(t)=0 are discarded, and the remaining samples are re-indexed to form a valid timeline.4.**Peak detection and suppression:** Define signal-specific thresholds$$\alpha =\{\alpha_{C},\alpha_{E},\alpha_{B},\alpha_{R^{1}},\ldots ,\alpha_{R^{j}}\}.$$For any signal $X\left(t\right)\in \{C\left(t\right),E\left(t\right),B\left(t\right),R^{j}\left(t\right)\}$, a peak is detected if$$|X\left(t\right)|>\alpha_{X}.$$Detected peaks are suppressed by zero replacement:X(t)←0.5.**Segment selection:** Define the set of beam-on indices$$T_{C}=\left\{\;t|C\left(t\right)>0\;\right\}.$$Let $t_{s}$ and $t_{e}$ denote the start and end of a contiguous beam-on interval. The set of maximal beam-on segments is$$\Gamma =\{\;\left[t_{s},t_{e}\right]\subseteq T_{C}|C\left(t\right)>0,\;\forall t\in \left[t_{s},t_{e}\right]\;\}.$$Short or unstable segments are discarded, and the remaining segments in Γ are used for modeling.

#### 3.2.1. STL Task

For the $j^{th}$ sensor, an independent model is trained to estimate the radiation value ${\hat{r}}_{t}^{j}$:

(1) $${\hat{r}}_{t}^{j}=f_{\theta }^{j}\left(c;e;b;r^{j}\right),\;j=1,2,3.$$

where ${\hat{r}}_{t}^{j}$ denotes the estimated radiation value for sensor *j* at time *t*; and $c=c_{t-m:t}$, $e=e_{t-m:t}$, $b=b_{t-m:t}$ represent the historical and current values of current, energy, and BLA over a sliding window of size *m*; $r^{j}=\left[r_{t-m:t-1}^{j},0\right]$ denotes the historical radiation values, where “0“ represents the current radiation value, to make all input variables the same length. The function $f_{\theta }^{j}$ is a model trained specifically for the $j^{th}$ sensor, and θ represents the set of parameters.

#### 3.2.2. MTL Task

In this context, an MTL model is trained to simultaneously estimate radiation values for all three radiation sensors at time *t*,

(2) $$\left[{\hat{r}}_{t}^{1},{\hat{r}}_{t}^{2},{\hat{r}}_{t}^{3}\right]=F_{\Theta }\left(c;e;b;r^{1};r^{2};r^{3}\right).$$

where $\left[{\hat{r}}_{t}^{1},{\hat{r}}_{t}^{2},{\hat{r}}_{t}^{3}\right]$ represent the estimated radiation values for sensors “rad48_p1”, “rad48_p2” and “rad29_p2” at the current time *t*, respectively. The function $F_{\Theta }\left(\cdot \right)$ denotes the proposed MTL model, parameterized by Θ.

### 3.3. Proposed MTL_TX Model

**Figure 5:**
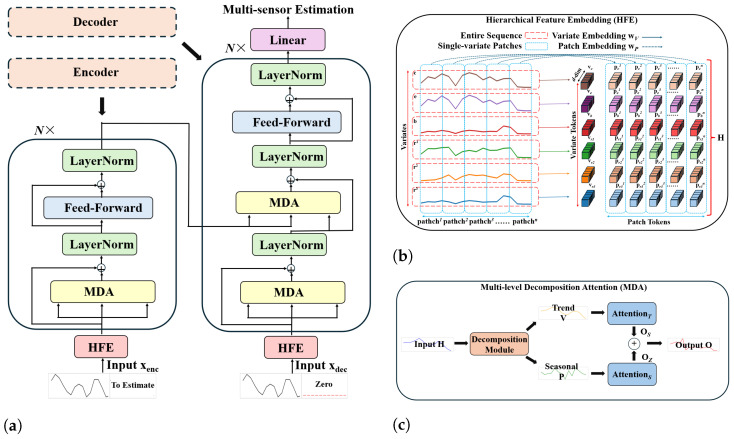
Overview of the proposed MTL_TX model. (**a**) The Transformer backbone of MTL, where the left half is the encoder and the right half is the decoder, both consisting of two novel components: hierarchical feature embedding (HFE) and multi-level decomposition attention (MDA). (**b**) Illustration of HFE, which transforms the input time series x into a hierarchical representation by embedding each variable’s entire sequence as variate tokens v and dividing individual variables into non-overlapping patches to generate patch tokens p. (**c**) Illustration of MDA, which decomposes the embedded input H into trend components V and seasonal components P, which are processed independently through attention mechanisms and then combined to generate output O. The model estimates radiation values for three sensors simultaneously.

Figure 5 illustrates the MTL_TX model, where the model first embeds input variables using the hierarchical feature embedding (HFE) module. The embedded vectors are then processed through the multi-level decomposition attention (MDA) module. Subsequently, the generative decoder utilizes the processed vectors to estimate radiation values for all sensors simultaneously. The HFE module combines global variate tokens and localized patch tokens to capture both long-term trends and short-term patterns, while the MDA module decomposes inputs into trend and seasonal components, thereby enhancing the model’s ability to capture complex temporal dependencies.

#### 3.3.1. Hierarchical Feature Embedding (HFE)

In the literature, iTransformer [18] introduced channel embedding (variate) to capture the global temporal features of time series, while PatchTST [17] proposed patch embedding to capture localized temporal patterns. In the HFE module, we integrate these two approaches to combine global and local features, capturing the hierarchical characteristics of radiation estimation.

Given the input matrix $x=[c;e;b;r^{1};r^{2};r^{3}]$, where $x\in R^{6\times m}$, a global variate token and multiple localized patch tokens are computed for each input variable separately. Using $c=c_{t-m:t}$ as an example, the global variate token $v_{c}$ is computed as

(3) $$v_{c}=cw_{V},$$

where $c\in R^{1\times m}$, and $w_{V}\in R^{m\times d}$ is a learnable weight matrix that projects c to a *d*-dimensional variate token. The input variable c is then divided into n=⌈m/p⌉ non-overlapping patches of length *p*, as follows:

(4) $$c_{\mathrm{patch}}^{i}=c_{t-m+(i-1)p:t-m+ip},i=1,2,\ldots ,n.$$

Each patch is mapped to a high-dimensional embedding space as

(5) $$p_{c}^{i}=c_{\mathrm{patch}}^{i}w_{P},$$

where $c_{\mathrm{patch}}^{i}\in R^{1\times p}$, and $w_{P}\in R^{p\times d}$ is a learnable weight matrix. The final embedding for c is obtained by concatenating the global variate token with all patch embeddings.

(6) $$h_{c}=\left[v_{c},p_{c}^{1},p_{c}^{2},\ldots ,p_{c}^{n}\right].$$

The same process is repeated for each input variable, and the final embedding input matrix, $H\in R^{6\times [(n+1)d]}$, is

(7) $$H=\left[\begin{array}{cccc} v_{c} & p_{c}^{1} & \ldots & p_{c}^{n}\\ v_{e} & p_{e}^{1} & \ldots & p_{e}^{n}\\ \vdots & \vdots & \ddots & \vdots \\ v_{r^{3}} & p_{r^{3}}^{1} & \ldots & p_{r^{3}}^{n} \end{array}\right].$$

#### 3.3.2. Multi-Level Decomposition Attention (MDA)

The MDA module consists of two attention mechanisms: the Trend Attention mechanism and the Seasonal Attention mechanism. Let H=[V,P], where

(8) $$V=\left[\begin{array}{c} v_{c}\\ v_{e}\\ \vdots \\ v_{r^{3}} \end{array}\right],\;\mathrm{and}\;P=\left[\begin{array}{ccc} p_{c}^{1} & \ldots & p_{c}^{n}\\ p_{e}^{1} & \ldots & p_{e}^{n}\\ \vdots & \ddots & \vdots \\ p_{r^{3}}^{1} & \ldots & p_{r^{3}}^{n} \end{array}\right].$$

The Trend Attention mechanism processes V to extract long-term dependencies and inter-variable relationships.

(9) $$O_{Z}={\mathrm{Attention}}_{Z}\left(V\right)=\mathrm{softmax}\left(\frac{Q_{Z}K_{Z}^{⊤}}{\sqrt{d_{u}}}\right)L_{Z},$$

(10) $$Q_{Z}=W_{Q}^{Z}V,\;K_{Z}=W_{K}^{Z}V,\;L_{Z}=W_{L}^{Z}V,$$

where $W_{Q}^{Z},W_{K}^{Z},W_{L}^{Z}\in R^{d\times d_{u}}$ are the learnable weight matrices for the query $Q_{Z}$, key $K_{Z}$, and value $L_{Z}$, and $d_{u}$ denotes the projected feature dimension. The Seasonal Attention mechanism processes P to capture localized variations.

(11) $$O_{S}={\mathrm{Attention}}_{S}\left(P\right)=\mathrm{softmax}\left(\frac{Q_{S}K_{S}^{⊤}}{\sqrt{d_{u}}}\right)L_{S},$$

(12) $$Q_{S}=W_{Q}^{S}P,\;K_{S}=W_{K}^{S}P,\;L_{S}=W_{L}^{S}P,$$

where $W_{Q}^{S},W_{K}^{S},W_{L}^{S}\in R^{d\times d_{u}}$ are the learnable weight matrices. The final output of the MDA module is obtained by combining the Trend and Seasonal Attention outputs as follows:

(13) $$O=O_{Z}+O_{S}.$$

MDA integrates global trends and localized seasonal features, enabling MTL_TX to effectively capture multivariate correlations and multi-level temporal dependencies.

#### 3.3.3. Inputs to Encoder and Decoder

The proposed MTL_TX adopts an encoder–decoder architecture in which radiation measurements are modeled as intrinsic system state variables rather than independent driving inputs. In accelerator environments, radiation levels exhibit coupled temporal dynamics influenced by beam current, energy, beam loss, and facility-specific operational conditions. Historical radiation measurements therefore encode the instantaneous system state from which future radiation levels can be inferred.

During training, the encoder receives the multivariate historical sequence

(14) $$X_{\mathrm{enc}}=\left[c;e;b;r^{1};r^{2};r^{3}\right]\in R^{6\times m},$$

The encoder aggregates beam-related inputs and radiation histories to form a global representation of the coupled system state and its long-range temporal dependencies.

The decoder input design is inspired by generative decoding in Informer [14]. Specifically, the decoder is fed with

(15) $$X_{\mathrm{dec}}=\left[r^{1};r^{2};r^{3}\right]\in R^{3\times m},$$

where $r^{j}=\left[r_{t-m:t-1}^{j},0\right]$ denotes the historical radiation measurements of the sensor *j*, augmented with a zero-valued placeholder that masks the radiation value at the current time step *t*. The decoder generates the estimated radiation values

(16) $$\hat{Y}=\left[{\hat{r}}_{t}^{1},{\hat{r}}_{t}^{2},{\hat{r}}_{t}^{3}\right],$$

Providing historical radiation measurements to the decoder serves two purposes in the MTL framework. First, they initialize the generative decoding process by supplying the most recent system state, enabling the modeling of short-term radiation dynamics that are not fully observable from beam parameters alone. Second, in the multi-output setting, the joint radiation history guides synchronized prediction across tasks, encouraging coherent multi-sensor estimation within a single forward pass. Beam-related variables (c, e, and b) are incorporated exclusively through encoder–decoder attention, allowing the decoder to focus on radiation state evolution conditioned on the encoded global context.

#### 3.3.4. Loss Functions

We utilize the mean squared error (MSE) loss with the Elastic Net regularization [29] to train the proposed model. Elastic Net incorporates $L_{1}$ regularization (LASSO), which induces sparsity by shrinking coefficients of less relevant features to zero, and incorporates $L_{2}$ regularization (Ridge), which prevents overfitting by penalizing large coefficients:

(17) $$L_{\mathrm{ElasticNet}}=\underset{t∼T}{E}∥r_{t}-{\hat{r}}_{t}{∥}_{2}^{2}+\lambda_{1}{∥\theta ∥}_{1}+\lambda_{2}{∥\theta ∥}_{2}^{2},$$

where the first term represents the MSE computed over the three sensors over the dataset, ${∥\theta ∥}_{1}$ and ${∥\theta ∥}_{2}^{2}$ denote the $L_{1}$ and $L_{2}$ regularization terms, respectively, $\lambda_{1}$ and $\lambda_{2}$ control the strength of the regularization to balance feature sparsity and smoothness.

### 3.4. Evaluation Metrics

In this study, we use Relative Absolute Error (RAE), Relative Squared Error (RSE) [30], Mean Absolute Error (MAE), Root Mean Squared Error (RMSE) [31], and the coefficient of determination ($R^{2}$) [32] to assess the performance of the proposed model. RAE represents the ratio of the absolute error to the actual value, while RSE measures the ratio of the squared error to the squared deviation of the actual value, both of which are used to assess the deviation between estimated and actual values. MAE computes the average of the absolute errors between the estimated and actual values, and RMSE quantifies the root mean square of the squared errors, representing the estimation errors produced by the model. For RAE, RSE, MAE, and RMSE, lower values indicate higher estimation accuracy.

The $R^{2}$ metric, also known as the coefficient of determination, evaluates the goodness-of-fit of the model. Higher $R^{2}$ values indicate better model performance and represent the ratio of the explained variance to the total variance in the regression task. The formulas for these metrics are defined as follows:

(18) $$\begin{array}{c} RAE=\frac{\sum_{t=1}^{T_{n}}\left|r_{t}-{\hat{r}}_{t}\right|}{\sum_{t=1}^{T_{n}}\left|\bar{r}-r_{t}\right|}\\ RSE=\frac{\sum_{t=1}^{T_{n}}{\left(r_{t}-{\hat{r}}_{t}\right)}^{2}}{\sum_{t=1}^{T_{n}}{\left(\bar{r}-r_{t}\right)}^{2}}\\ MAE=\frac{1}{T}\sum_{t=1}^{T}\left|r_{t}-{\hat{r}}_{t}\right|\\ RMSE=\sqrt{\frac{1}{T}\sum_{t=1}^{T}{\left(r_{t}-{\hat{r}}_{t}\right)}^{2}}\\ R^{2}=1-\frac{\sum_{t=1}^{T_{n}}{\left(r_{t}-{\hat{r}}_{t}\right)}^{2}}{\sum_{t=1}^{T_{n}}{\left(\bar{r}-r_{t}\right)}^{2}} \end{array}$$

where $\bar{r}$ represents the mean of actual values. These metrics provide a comprehensive evaluation of the model’s accuracy and robustness in estimating radiation values.

### 3.5. Choice of Competing Models

We compare MTL_TX with several baseline models, including STL models proposed in [6], such as linear regression (LR) [33], random forest (RF) [34], multilayer perceptron (MLP) [35], support vector regressor (SVR) [36], STL CNN [37], STL LSTM [38], and STL Vanilla Transformer (STL_TX). Each STL model was optimized using the Optuna optimizer [39], a Bayesian hyperparameter optimization framework designed to identify optimal configurations efficiently. For the RF model, grid search was employed to determine the best parameter settings. We also compared the proposed model with the MTL bypass-path framework, where CNN and LSTM are selected as backbones.

## 4. Experimental Setups

### 4.1. Experiments

To validate the effectiveness of the proposed MTL framework for time-series radiation estimation for JLab-specific tasks, we conducted the following four experiments.

#### 4.1.1. Hyperparameter Optimization

In the first experiment, we performed hyperparameter optimization for STL_TX and MTL_TX using JLab’s 2018 dataset. STL_TX focuses on estimating radiation values exclusively for the “rad48_p1” sensor using data from Hall A sensors, while MTL_TX simultaneously estimates radiation values for three sensors: “rad48_p1,” “rad48_p2,” and “rad29_p2”. We employed the Optuna optimizer [39] to identify the optimal hyperparameter configurations, aiming to maximize the $R^{2}$ score on the training data. Both models utilized a historical window size of m=15. To ensure fairness, we adjusted the configurations to maintain a similar parameter count for both models.

#### 4.1.2. Comparison Study with Competing Models

As illustrated in Figure 2, the recorded data from 2018 were divided into three segments. The second segment was used to train all competing models due to the inclusion of significant variations in input current. The first and third segments were then used for model evaluation. All competing models underwent a parameter search using Optuna to determine optimal architectures. The MTL LSTM and MTL CNN models from previous work retained their original configurations as reported in [6]. All models were configured with the same input settings, leveraging historical data from multiple sensors to estimate the current radiation values for each sensor.

#### 4.1.3. Model Generalization Testing on Data from Different Years

The three data segments from 2018 exhibit similar distribution characteristics and are highly correlated. Consequently, evaluating model performance solely on these segments is insufficient to demonstrate the models’ robustness. To further assess generalization capabilities, all trained competing models were tested on archived data from the years 2016, 2017, and 2019. As shown in Table 2, the datasets from these years comprise one segment due to substantial noise, with only relatively clean segments included for testing. Evaluating performance on these datasets provides a comprehensive assessment of the robustness of the competing models.

#### 4.1.4. Ablation Study

##### Ablation Study for Different Architectures

The architecture ablation study evaluates the roles of the encoder, decoder, and cross-sensor information modeling. Three architectural variants were considered. (1) Encoder-only: the model directly maps beam-related variables, including injected current c, energy e, and BLA b, together with cross-sensor historical radiation sequences $\left(r^{1},r^{2},r^{3}\right)$, to estimate the current radiation values. (2) Encoder–decoder without cross-sensor radiation in the encoder: the encoder is restricted to beam-related inputs (c,e,b), while the decoder is conditioned on historical radiation sequences to perform estimation. (3) Proposed full encoder–decoder: the encoder jointly models beam conditions and cross-sensor radiation histories, and the decoder performs conditioned temporal generation based on radiation sequences.

##### Ablation Study for Different Decoder Inputs

The decoder input ablation study investigates the role of historical radiation conditioning in the decoder while keeping the encoder identical to the full MTL_TX configuration. Three decoder input settings are evaluated.

(1) Full radiation conditioning:

$$X_{\mathrm{dec}}^{\left(1\right)}=\left[r^{1};r^{2};r^{3}\right]\in R^{3\times m},$$

where $r^{j}$ denotes the historical radiation sequence of sensor *j*.

(2) Zero-sequence conditioning:

$$X_{\mathrm{dec}}^{\left(2\right)}=\left[0;0;0\right]\in R^{3\times m},$$

which removes radiation information while preserving temporal structure.

(3) Zero-token conditioning:

$$X_{\mathrm{dec}}^{\left(3\right)}=\left[0,0,0\right]\in R^{3\times m},$$

which collapses the decoder input to a constant non-informative sequence.

For all configurations, the model outputs the multi-sensor radiation estimates $\left[r_{t}^{1},r_{t}^{2},r_{t}^{3}\right]$ at the current time step *t*.

##### Ablation Study for the Components in the Proposed Method

The component-wise ablation study evaluates the contribution of individual modules in MTL_TX. The baseline model was a Vanilla Transformer, serving as a reference for performance comparison. The proposed components were incrementally added to the baseline model. First, Elastic Net was introduced into the baseline model to enforce sparsity and mitigate overfitting. Next, the HFE module replaced the standard temporal embedding, introducing high-dimensional representations that capture both global and local sequence features. Finally, the MDA module was integrated to construct the fully optimized MTL_TX model, enabling the decomposition of input sequences into trend and seasonal components within the attention mechanism. Notably, MDA is designed to complement HFE, as the hierarchical structure provided by HFE enhances the effectiveness of decomposition-based attention.

### 4.2. Implementation Details

All models in this study were implemented using the Keras 3.8.0 [40] and the PyTorch 2.6.0 [41] frameworks. Hyperparameter optimization was performed using the Optuna optimizer, with 300 trials used to identify an optimal historical window size of m=15. For all deep learning models, the Adam optimizer was used with an initial learning rate of 0.001, a weight decay of 1 ×$10^{-5}$, a batch size of 256, and a maximum of 100 epochs. Early stopping was applied with a patience of 10 epochs to prevent overfitting. MTL_TX was configured with four attention heads, a hidden dimension of 128, and a feedforward network dimension of 256. A dropout rate of 0.1 was used to enhance generalization. Elastic Net regularization was applied with α=0.1 to control the overall regularization strength, while the $L_{1}$ scaling factor ρ=0.5 was used to ensure a balanced contribution from $L_{1}$ (sparsity) and $L_{2}$ (smoothness) regularization. All experiments were conducted on the high-performance computing cluster at Old Dominion University, utilizing NVIDIA A100 GPUs to accelerate training.

## 5. Results

### 5.1. Hyperparameter Optimization Results

**Figure 6:**
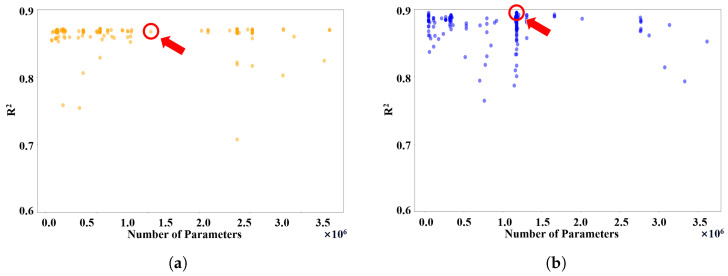
Hyperparameter optimization results for (**a**) STL_TX and (**b**) MTL_TX over 300 trials using the Optuna optimizer. The X-axis represents the number of parameters in each trial, while the Y-axis corresponds to the training $R^{2}$ score of the respective model. The red circles denote the final selected STL_TX and MTL_TX configurations, which achieve the best performance while maintaining a similar parameter count.

Figure 6 illustrates the hyperparameter search results for STL_TX and MTL_TX. The X-axis represents the number of parameters in each trial, while the Y-axis denotes the corresponding $R^{2}$ score. On average, the MTL_TX model outperformed the STL_TX model over 300 trials. For a fair comparison, we selected STL_TX and MTL_TX configurations with approximately 12,000 parameters for subsequent experiments, as highlighted in Figure 6. Notably, trials with similar parameter counts exhibited variations in $R^{2}$ scores due to random parameter initialization, thereby leading to different local optima. We evaluated STL_TX and MTL_TX on the first and third segments of the 2018 archived dataset, with the results presented in Table 3. MTL_TX achieved the best performance, with an RSE of 22.0956%, RAE of 32.4656%, MAE of 0.1464, RMSE of 0.2353 and an $R^{2}$ score of 0.8584, significantly outperforming STL_TX.

### 5.2. Comparative Results on Data Collected in 2018

Table 4 presents the average test results for all competing models on the first and third segments of the 2018 dataset. All models were trained on the second segment of the data. MTL_TX achieved the best performance across all metrics. Notably, it significantly outperformed the MTL LSTM model of our previous work, demonstrating clear improvements. It is also worth mentioning that STL_TX performed comparably to the MTL LSTM model, further highlighting the remarkable capability of the Transformer architecture in handling time-series modeling. Additionally, MTL models consistently outperformed STL models, showcasing the advantage of the MTL approach in capturing valuable inter-sensor correlations. This is particularly crucial for JLab’s radiation estimation tasks, where data collected from multiple sensors exhibits inherent dependencies.

### 5.3. Generalization Results on Data from Different Years

Table 5 presents the average test results of all competing models on datasets from 2016, 2017, and 2019, where all models were trained on the second segment of the 2018 data. MTL_TX achieved the best performance, with an average RSE of 37.2067%, RAE of 21.2411%, MAE of 0.1407, RMSE of 0.2263, and an $R^{2}$ score of 0.8831, demonstrating its superior generalization capability in estimating radiation values across different years. This is particularly critical for JLab’s radiation estimation tasks, where data characteristics can vary significantly due to operational or environmental factors. The findings further confirm that the MTL framework effectively leverages inter-sensor correlations and temporal dependencies, enabling reliable estimation on unseen datasets.

### 5.4. Ablation Study Results

#### 5.4.1. Architecture Ablation Study

Table 6 summarizes the architecture ablation results. The encoder-only model achieves moderate performance, with an $R^{2}$ of 0.5859, indicating that joint modeling of beam-related variables and cross-sensor radiation history captures part of the radiation state. In contrast, removing cross-sensor radiation inputs from the encoder leads to a substantial performance degradation ($R^{2}=0.1670$), suggesting that beam-related signals alone are insufficient for reliable radiation estimation. The proposed full encoder–decoder architecture achieves the best performance ($R^{2}=0.8584$), demonstrating that accurate estimation requires both cross-sensor state representation in the encoder and conditioned temporal generation in the decoder.

#### 5.4.2. Decoder Input Ablation Study

Table 7 reports the results of the decoder input ablation study. When historical radiation information is removed from the decoder, performance degrades substantially. Both the zero-token and zero-sequence configurations yield limited accuracy, with $R^{2}$ values of 0.5802 and 0.6127, respectively. In contrast, full historical radiation conditioning achieves significantly better performance ($R^{2}=0.8584$). The relatively small gap between zero-token and zero-sequence settings indicates that preserving temporal structure alone is insufficient without meaningful radiation-based state information. These results confirm that historical cross-sensor radiation conditioning is essential for effective decoder operation.

#### 5.4.3. Component-Wise Ablation Study

Table 8 presents the results of this component-wise ablation study. The results demonstrate that the incremental addition of each component progressively improved model performance. The final MTL model with all components achieved the best results, reaching an $R^{2}$ of 0.8584. Notably, the inclusion of the HFE component provided the most significant performance improvement, underscoring the effectiveness of combining variate and patch embeddings for the data collected at JLab. Our results confirm the effectiveness of the proposed components within the MTL_TX framework. Each component contributes to the overall improvement of the model, with HFE and MDA playing critical roles in enhancing the handling of multivariate time-series data.

### 5.5. Case Study on Radiation Estimation

**Figure 7:**
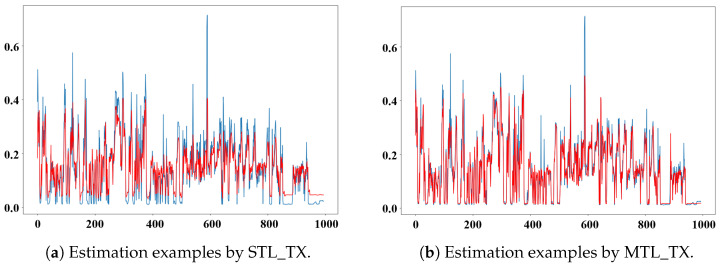
Estimations by STL_TX and MTL_TX for the second segment of the “rad48_p1” sensor data collected in 2018.

**Figure 8:**
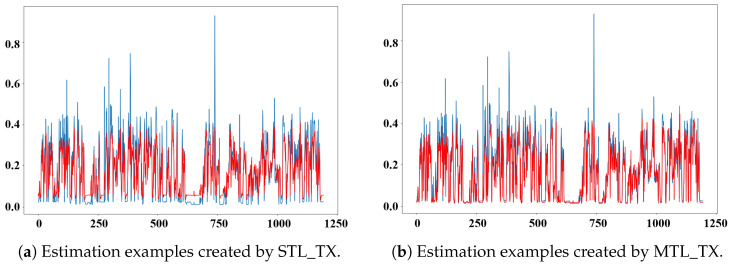
Estimations by STL_TX and MTL_TX for the first segment of the “rad48_p1” sensor data collected in 2018.

To visually evaluate the radiation estimation performance of the MTL_TX model, we compared the estimations of STL_TX and MTL_TX using data from the “rad48_p1” sensor. Figure 7 illustrates the training results of both models on the second segment of the 2018 data from the “rad48_p1” sensor. The red curve represents the radiation values estimated by the models, while the blue curve shows the ground truth. MTL_TX clearly demonstrates superior performance, particularly in regions where the radiation level approaches zero and at sharp local extrema. Figure 8 shows the testing results of the two Transformer models on the first segment of the 2018 data for the same sensor. MTL_TX continues to outperform the STL_TX, providing better overall fits. In particular, MTL_TX demonstrates improved peak fitting accuracy and reduced deviations around near-zero radiation levels, indicating stronger robustness under low-signal and rapidly varying conditions. In near-zero regimes, STL_TX tends to collapse toward mean-dominated predictions, whereas MTL_TX maintains stable tracking behavior. This advantage can be attributed to the multi-task learning mechanism, in which correlated radiation sensors jointly contribute to a shared state representation, thereby alleviating the low signal-to-noise challenges encountered by single-task models. However, both models fail to accurately estimate a sudden peak in the data. Further investigation revealed that this peak was caused by a recording error, which introduced an abrupt jump that cannot be effectively learned by the models.

**Figure 9:**
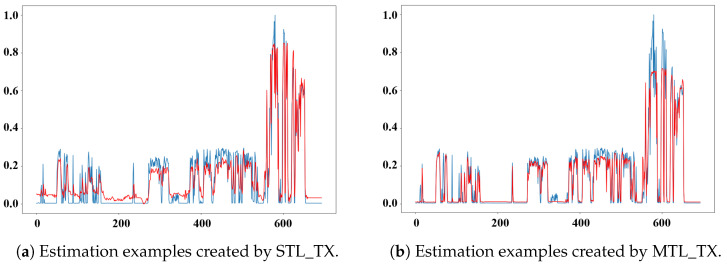
Estimations by STL_TX and MTL_TX for the first segment of the “rad48_p1” sensor data collected in 2016.

Figure 9 presents estimation examples from both trained STL_TX and trained MTL_TX on data collected in 2016. It can be observed that the data collected in 2016 exhibit a distinctly different distribution pattern, particularly with noticeable increases in radiation values during certain periods. The estimation results of STL_TX show significant deviations from the actual radiation data, indicating poor generalization to unseen datasets. In contrast, MTL_TX demonstrates superior generalization capability, effectively adapting to distributional changes in the 2016 dataset.

In some peak regions of the dataset (Figure 9), STL_TX appears to capture sharper peaks than MTL_TX. This behavior can be attributed to the fact that STL_TX is trained to fit a single radiation sensor and is therefore more sensitive to large-magnitude variations, which can lead to more accurate fitting of isolated peaks. In contrast, MTL_TX enforces cross-sensor consistency through shared representations, producing smoother and more robust estimates that suppress sensor-specific fluctuations unsupported by other measurements. Overall, MTL_TX consistently outperforms STL_TX, achieving excellent performance across datasets from different years. This further validates the effectiveness of the proposed components within MTL_TX.

## 6. Discussions

This study aims to develop a Transformer-based MTL model to simultaneously estimate radiation values for sensors within the JLab facility. Two novel components, hierarchical feature embedding (HFE) and multi-level decomposition attention (MDA), were integrated into the proposed model, and it was compared against multiple competing models to evaluate its estimation performance. All of the competing models were tested on datasets collected by JLab from 2016 to 2019. MTL_TX demonstrated the best overall estimation performance. On the 2018 dataset, it achieved average scores of RAE = 32.4656%, RSE = 22.0956%, MAE = 0.1464, RMSE = 0.2353, and $R^{2}$ = 0.8584. In addition, it exhibited superior generalization performance on unseen data from other years, with average scores of RAE = 31.2067%, RSE = 21.2411%, MAE = 0.1407, RMSE = 0.2263, and $R^{2}$ = 0.8831.

**Figure 10:**
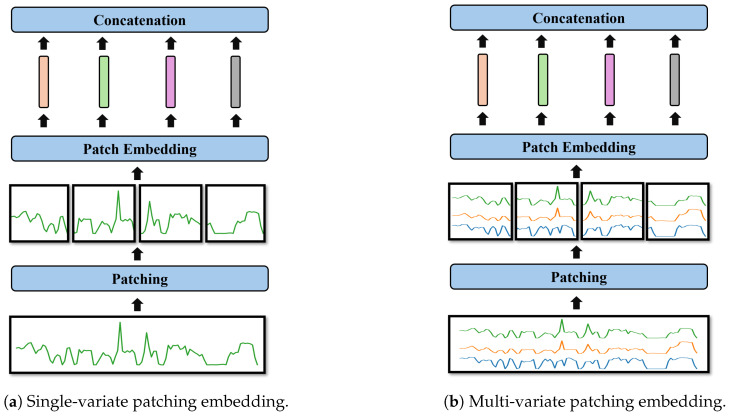
Variate embedding comparison. (**a**) Single-variate patching embedding, where each variable is independently embedded; (**b**) Multi-variate patching embedding, where multiple variables are jointly embedded to form patches containing cross-variable information.

Compared to STL models, MTL_TX significantly enhances its own ability to extract latent correlations and features from multivariate time-series data, as shown in Figure 10. The STL models are limited to feature extraction for a single radiation sensor and cannot be directly generalized to achieve high-accuracy radiation estimation for other sensors. Each radiation sensor requires a separate STL model, leading to an inefficient deployment strategy that consumes substantial computational resources and increases costs. The primary advantage of the MTL framework lies in its ability to process data from all sensors deployed around Hall A and simultaneously estimate radiation values for multiple sensors. By leveraging the MTL approach, the proposed model provides significant advantages, including reduced computational redundancy, enhanced feature extraction, and improved estimation accuracy.

All three components—ElasticNet, HFE, and MDA—play a crucial role in the proposed model. ElasticNet introduces constraints to mitigate overfitting. HFE integrates variate embedding and univariate patch embedding to capture both global and local information from the input time-series data. The global embeddings provide a comprehensive view of the dataset, while the local embeddings capture localized temporal information within each individual variable channel. Both embeddings enhance the MDA component’s ability to learn multivariate correlations effectively. Results from the ablation study clearly demonstrate the significant contributions of ElasticNet (improving $R^{2}$ from 0.7792 → 0.8086), HFE ($R^{2}$ 0.8068 → 0.8377), and MDA ($R^{2}$ 0.8377 → 0.8584).

The novelty of this work lies in integrating Transformer-based multi-task learning into radiation monitoring by formulating the problem as a multi-sensor estimation task rather than radiation transport physics modeling. The proposed MTL_TX framework targets robust radiation estimation under noisy, incomplete, and heterogeneous sensor conditions encountered in operational accelerator environments. By jointly modeling multiple radiation sensors within a unified framework, MTL_TX enables effective cross-sensor information sharing and consistent state estimation that cannot be achieved by independent single-task models.

From a deployment perspective, the final MTL_TX configuration contains approximately $1.2\times 10^{4}$ trainable parameters and performs radiation estimation through a single forward pass without iterative decoding. In our implementation, a single forward pass requires approximately 10.596 ms. Given the hourly sampling interval, the inference cost is orders of magnitude smaller than the data acquisition interval, making it negligible in practice. As a result, MTL_TX readily satisfies near-real-time requirements while offering reliable, scalable, and low-overhead deployment for radiation monitoring systems.

MTL_TX has a substantial number of parameters, and this significantly contributes to its superior performance. However, we observed that increasing the number of encoder and decoder layers only yielded a slight improvement in performance while dramatically increasing the number of parameters and the training time. To balance model efficiency and performance, MTL_TX in this study comprises two encoder layers and two decoder layers. With advancements in hardware technology and increasing computational power of graphics processing units (GPUs), training time is expected to decrease significantly in the future [42]. Future work may involve adjusting the model architecture to fully exploit the potential of evolving hardware capabilities.

We conducted extensive experiments on both model architecture and input design to investigate the impact of structural choices and information sources on radiation estimation performance. Specifically, we explored encoder-only configurations, encoder–decoder variants with restricted information flow, and multiple decoder input conditioning strategies. Across all evaluations, the full encoder-decoder architecture consistently achieved the best performance. The results indicate that effective radiation estimation requires both explicit cross-sensor state representation in the encoder and conditioned temporal generation in the decoder. Cross-sensor radiation information plays a critical role in capturing shared operational states and inter-sensor dependencies, which cannot be reliably inferred from beam-related variables alone. These findings highlight the importance of jointly modeling beam conditions and cross-sensor radiation histories within a unified framework for robust multi-sensor radiation estimation.

This study has several limitations. First, the performance of MTL_TX depends on the quality and availability of archived multi-sensor data from JLab. The historical data is sparse, noisy, and peak-containing. We utilized simple methods to eliminate these peaks as anomalies; more advanced techniques will be explored for preprocessing. Second, this study used hourly-sampled data for model training and testing. However, JLab also collects data with finer temporal resolution, such as 10 min or 1 min intervals, which capture more detailed radiation fluctuations. Future work will explore multiple temporal resolutions to evaluate the adaptability of MTL_TX to finer-grained time scales. Third, the proposed model was validated using sensor data from JLab’s accelerator facility only. We will apply it to datasets from other DOE accelerator facilities to further evaluate its generalization capability.

## 7. Conclusions

We propose a Transformer-based MTL model for estimating radiation values from multiple sensors deployed at JLab’s accelerator facility. We introduce two innovative components, including hierarchical feature embedding and multi-level decomposition attention, to boost the performance of the proposed model. The proposed model was thoroughly evaluated against several baseline models, including both single-task and multi-task approaches, using archived radiation data collected from 2016 to 2019 at JLab. Experimental results demonstrated that the proposed model achieved superior performance in estimating radiation values, with the highest $R^{2}$ and the lowest error metrics on both the dataset from the training year and unseen datasets from other years. In conclusion, the proposed MTL_TX model represents a significant advancement in JLab radiation estimation applications. Future work will focus on further enhancing the model’s capabilities and validating its effectiveness across diverse datasets and operational scenarios.

## Figures and Tables

**Table 1:** Specifications and usage of sensors located around Hall “A” at JLab.

Sensor Type	Sensor Name	Model Usage
Current	IBC1H04CRCUR2	Input
Energy	MMSHLAE	Input
BLA	IBCBS05CLOSS	Input
Gamma	rad48_p1, rad43_p1, rad29_p1	Input & Output
Neutron	rad48_p2, rad43_p2, rad29_p2	Input & Output

**Table 2:** Hourly radiation series collected at JLab in different years of various segments.

Year	No. of Samples	Segment 1	Segment 2	Segment 3
2016	8783	800–1700		
2017	8759	1000–1700		
2018	8759	200–1500	1900–3000	6400–8000
2019	8759	1000–1900		

**Table 3:** Testing results of STL_TX and MTL_TX on the dataset from the year 2018.

Model	RAE (%) ↓	RSE (%) ↓	MAE ↓	RMSE ↓	R^2^ ↑
STL_TX	36.2467	24.6644	0.1635	0.2628	0.7823
**MTL_TX**	**32.4656**	**22.0956**	**0.1464**	**0.2353**	**0.8584**

**Table 4:** Testing results of all competing models on the dataset from the year 2018.

Model	RAE (%) ↓	RSE (%) ↓	MAE ↓	RMSE ↓	R^2^ ↑
RF	47.8589	32.5933	0.2156	0.3467	0.5961
LR	38.3378	26.0767	0.1728	0.2777	0.7426
MLP	38.5878	26.2611	0.1740	0.2798	0.7354
SVR	37.8222	25.7333	0.1706	0.2741	0.7521
STL_CNN	37.6189	25.5944	0.1696	0.2726	0.7559
STL_LSTM	37.8600	25.7578	0.1707	0.2745	0.7479
MTL_CNN	36.8533	25.0789	0.1662	0.2671	0.7689
MTL_LSTM	35.6344	24.1978	0.1604	0.2579	0.7993
STL_TX	36.2467	24.6644	0.1635	0.2628	0.7823
**MTL_TX**	**32.4656**	**22.0956**	**0.1464**	**0.2353**	**0.8584**

**Table 5:** Testing results of all competing models on datasets from the years 2016 to 2019.

Model	RAE (%) ↓	RSE (%) ↓	MAE ↓	RMSE ↓	R^2^ ↑
RF	70.6356	48.0622	0.3187	0.5123	0.4887
LR	45.2811	30.8100	0.2041	0.3281	0.6675
MLP	50.3078	34.2344	0.2268	0.3645	0.6254
SVR	46.4956	31.6289	0.2094	0.3367	0.6666
STL_CNN	43.3478	29.4789	0.1954	0.3142	0.6880
STL_LSTM	46.4867	31.6300	0.2094	0.3367	0.6539
MTL_CNN	44.1033	29.9889	0.1986	0.3193	0.7176
MTL_LSTM	42.7933	29.0778	0.1927	0.3101	0.7246
STL_TX	35.0022	23.7856	0.1573	0.2530	0.8027
**MTL_TX**	**31.2067**	**21.2411**	**0.1407**	**0.2263**	**0.8831**

**Table 6:** Ablation Study on Model Architecture.

Model	Encoder	Decoder	Cross-Sensor	RAE (%) ↓	RSE (%) ↓	MAE ↓	RMSE ↓	$R^{2}$↑
MTL_TX	✓		✓	46.19	63.00	0.4252	1.0239	0.5859
MTL_TX	✓	✓		79.80	85.49	0.6712	1.3544	0.1670
**MTL_TX**	✓	✓	✓	**32.47**	**22.10**	**0.1464**	**0.2353**	**0.8584**

**Table 7:** Decoder Input Ablation Results on the 2018 Test Segment.

Model	Decoder Input	RAE (%) ↓	RSE (%) ↓	MAE ↓	RMSE ↓	R^2^ ↑
MTL_TX	Zero-token	52.7694	62.4777	0.4633	1.0051	0.5802
MTL_TX	Zero-sequence	51.8136	61.0044	0.4674	0.9923	0.6127
MTL_TX	**Full radiation**	**32.4656**	**22.0956**	**0.1464**	**0.2353**	**0.8584**

**Table 8:** Ablation study results.

Model	ElasticNet	HFE	MDA	RAE (%) ↓	RSE (%) ↓	MAE ↓	RSME ↓	R^2^ ↑
MTL_TX				36.3411	24.7433	0.1639	0.2634	0.7792
MTL_TX	✓			35.0733	23.8511	0.1582	0.2541	0.8068
MTL_TX	✓	✓		33.4022	22.7433	0.1505	0.2421	0.8377
**MTL_TX**	✓	✓	✓	**32.4656**	**22.0956**	**0.1464**	**0.2353**	**0.8584**

## Data Availability

The data presented in this study are not publicly available due to project-related confidentiality and ongoing collaborative agreements, but may be made available from the corresponding author upon reasonable request.

## References

[1] Wallo A., Domotor S., Vazquez G. (2006). US Department of Energy policies, directives, and guidance for radiological control and release of property. Health Phys..

[2] Liu C.H., Gu J.C., Yang M.T. (2021). A simplified LSTM neural networks for one day-ahead solar power forecasting. IEEE Access.

[3] Liu Z., Sullivan C.J. (2019). Prediction of weather induced background radiation fluctuation with recurrent neural networks. Radiat. Phys. Chem..

[4] Jin H., Ma H., Butala M.D., Liu E.X., Li E.P. (2019). EMI radiation prediction and structure optimization of packages by deep learning. IEEE Access.

[5] Cho C., Kwon K., Wu C. (2022). On weather data-based prediction of gamma exposure rates using gradient boosting learning for environmental radiation monitoring. Sensors.

[6] Zhang H., Stavola A., Ferguson H., Budavari B., Kwan C., Wu H., Li J. (2024). Deep Multi-task Learning Models for Radiation Estimation at High Energy Accelerator Facility. IEEE Trans. Nucl. Sci..

[7] Vaswani A., Shazeer N., Parmar N., Uszkoreit J., Jones L., Gomez A.N., Kaiser L., Polosukhin I. (2017). Attention is all you need. Advances in Neural Information Processing Systems 30: Annual Conference on Neural Information Processing Systems 2017, Long Beach, CA, USA, 4–9 December 2017.

[8] Leemann C.W., Douglas D.R., Krafft G.A. (2001). The continuous electron beam accelerator facility: CEBAF at the Jefferson Laboratory. Annu. Rev. Nucl. Part. Sci..

[9] Burkert V.D. (2018). Jefferson Lab at 12 GeV: The science program. Annu. Rev. Nucl. Part. Sci..

[10] Devlin J., Chang M.W., Lee K., Toutanova K. (2018). BERT: Pre-training of deep bidirectional transformers for language understanding. arXiv.

[11] Brown T.B., Mann B., Ryder N., Subbiah M., Kaplan J.D., Dhariwal P., Neelakantan A., Shyam P., Sastry G., Askell A. (2020). Language models are few-shot learners. Adv. Neural Inf. Process. Syst..

[12] Dosovitskiy A., Beyer L., Kolesnikov A., Weissenborn D., Zhai X., Unterthiner T., Dehghani M., Minderer M., Heigold G., Gelly S. (2020). An image is worth 16 × 16 words: Transformers for image recognition at scale. arXiv.

[13] Dong L., Xu S., Xu B. (2018). Speech-transformer: A no-recurrence sequence-to-sequence model for speech recognition. 2018 IEEE International Conference on Acoustics, Speech and Signal Processing (ICASSP).

[14] Zhou H., Zhang S., Peng J., Zhang S., Li J., Xiong H., Zhang W. (2021). Informer: Beyond efficient transformer for long sequence time-series forecasting. Proc. AAAI Conf. Artif. Intell..

[15] Wu H., Xu J., Wang J., Long M., Jiang X. (2021). Autoformer: Decomposition transformers with auto-correlation for long-term series forecasting. Adv. Neural Inf. Process. Syst..

[16] Zhou T., Ma Z., Wen Q., Wang X., Sun L., Jin R. (2022). FEDformer: Frequency enhanced decomposed transformer for long-term series forecasting. Proceedings of the 39th International Conference on Machine Learning (ICML).

[17] Nie Y., Xu J., Yuan F., Wang G., Liu X., He G. (2022). Time-series representation learning via patch-based modeling: A unified view and comparative analysis. arXiv.

[18] Liu C., Xu Y., Liu X., Yang Y., Li J. (2023). iTransformer: Transforming long time-series forecasting with variate tokens. arXiv.

[19] Wei J., Zhang H., Stavola A., Budavari B., Kwan C., Wu H., Li J. (2024). Optimizing Transformer-based Models for Radiation Estimation at High Energy Accelerator Facility. Proceedings of the 2024 IEEE 15th Annual Ubiquitous Computing, Electronics & Mobile Communication Conference (UEMCON).

[20] Cao-Xue J., Zhang H., Stavola A., Budavari B., Kwan C., Wu H., Li J. (2024). Improved Radiation Estimation at High Energy Facility for Public Health. Proceedings of the 2024 IEEE 15th Annual Ubiquitous Computing, Electronics & Mobile Communication Conference (UEMCON).

[21] Xu T., Zhang K., Han J., Li C., He L. (2021). Anomalyformer: Time series anomaly detection with variable-length reconstruction via robust transformer. Adv. Neural Inf. Process. Syst..

[22] Lim B., Arık S.O., Loeff N., Pfister T. (2021). Temporal fusion transformers for interpretable multi-horizon time series forecasting. Int. J. Forecast..

[23] Li J., Xu Y., Wang Y., Tang C., Jiang S., Xu Y. (2022). IoTformer: Efficient and interpretable transformer for industrial IoT applications. arXiv.

[24] Caruana R. (1997). Multitask learning. Mach. Learn..

[25] Zhang W., Wang L., Liu Y., Zhang B. (2022). MTTrans: Multitask transformer for time-series classification and forecasting. Proceedings of the 2022 IEEE International Conference on Data Mining (ICDM).

[26] Zhang Y., Zheng K., Jiang Y., Wu J. Crossformer: Transformer utilizing cross-dimension dependency for multivariate time series forecasting. Proceedings of the ICLR 2023.

[27] Jaegle A., Gimeno F., Brock A., Zisserman A., Vinyals O., Carreira J. Perceiver: General perception with iterative attention. Proceedings of the 38th International Conference on Machine Learning (ICML).

[28] Koo B., Sung I., Lee S., Kim S. (2025). Transcriptome Transformer: Improving patient survival prediction via multitask learning of transcriptomic and clinical features. Brief. Bioinform..

[29] Zou H., Hastie T. (2005). Regularization and variable selection via the elastic net. J. R. Stat. Soc. Ser. B Stat. Methodol..

[30] Botchkarev A. (2018). Performance metrics (error measures) in machine learning regression, forecasting and prognostics: Properties and typology. arXiv.

[31] Chicco D., Warrens M.J., Jurman G. (2021). The coefficient of determination R-squared is more informative than SMAPE, MAE, MAPE, MSE and RMSE in regression analysis evaluation. PeerJ Comput. Sci..

[32] Di Bucchianico A. (2008). Coefficient of determination (R^2^). Encycl. Stat. Qual. Reliab..

[33] Vapnik V., Lerner J. (2003). Least Squares Support Vector Machines. J. Mach. Learn. Res..

[34] Breiman L. (2001). Random Forests. Mach. Learn..

[35] Rumelhart D.E., Hinton G.E., Williams R.J. (1986). Learning Representations by Back-Propagating Errors. Nature.

[36] Cortes C., Vapnik V. (1995). Support Vector Networks. Mach. Learn..

[37] Krizhevsky A., Sutskever I., Hinton G. (2012). ImageNet Classification with Deep Convolutional Neural Networks. Proc. Adv. Neural Inf. Process. Syst..

[38] Hochreiter S., Schmidhuber J. (1997). Long Short-Term Memory. Neural Comput..

[39] Akiba T., Sano S., Yanase T., Ohta T., Koyama M. (2019). Optuna: A next-generation hyperparameter optimization framework. Proceedings of the 25th ACM SIGKDD International Conference on Knowledge Discovery & Data Mining.

[40] Ketkar N., Ketkar N. (2017). Introduction to keras. Deep Learning with Python: A Hands-On Introduction.

[41] Paszke A., Gross S., Massa F., Lerer A., Bradbury J., Chanan G., Killeen T., Lin Z., Gimelshein N., Antiga L. (2019). Pytorch: An imperative style, high-performance deep learning library. Advances in Neural Information Processing Systems 32.

[42] Deng Y. (2019). Deep learning on mobile devices: A review. Proceedings of the Mobile Multimedia/Image Processing, Security, and Applications 2019.

